# Changes in self-reflective thinking level in writing and educational needs of medical students: A longitudinal study

**DOI:** 10.1371/journal.pone.0262250

**Published:** 2022-01-21

**Authors:** Kwi Hwa Park, Bee Sung Kam, So Jung Yune, Sang Yeoup Lee, Sun Ju Im

**Affiliations:** 1 Department of Medical Education, Gachon University College of Medicine, Incheon, South Korea; 2 Department of Medical Education, Pusan National University School of Medicine, Busan, South Korea; University of Hradec Kralove: Univerzita Hradec Kralove, CZECH REPUBLIC

## Abstract

**Introduction:**

The purpose of this study was to longitudinally examine the change in understanding of the self-reflection method, reflective thinking, and writing attitude and perception. Moreover, we investigated students’ educational needs and methods regarding self-reflection.

**Methods:**

The subjects were 117, who were in the pre-medical course in 2017 and were promoted to the medical course in 2019. Questions concerning students’ understanding of self-reflection methods, their attitude and perception of reflective writing, and educational needs and methods regarding self-reflection were self-developed. For students’ reflective thinking level in writing, we used the approach developed by Galvez-Martin, Bowman, and Morrison and adapted by Kwon. For dada analysis, χ^2^ test, t-test, frequency analysis was used.

**Results:**

We found that students’ level of understanding regarding self-reflection methods increases slightly, but not significant (χ^2^ = 2.238, p>0.05). There was no significant change in the level of reflective thinking in writing (χ^2^ = 8.003, p>0.05). The students’ attitude toward reflective writing decreased in the medical course than in the pre-medical course (t = 3.475, p<0.001). The perception that reflective writing was helpful for individual improvement decreased during the medical course (t = 4.931, p<0.001). The need for self-reflection education increased in the medical course (t = -2.659, p<0.001). They preferred implementation in the first year of the medical course as an instructor-led special lecture.

**Conclusion:**

Self-reflective ability is not naturally developed as students’ progress through grade levels. Educational intervention is needed to help students understand approaches to self-reflection and its importance in enabling them to develop their abilities as well as to participate actively in reflective writing.

## Introduction

Medical education is designed to aid the development of professional behavior in medical students. Professional behavior assumes that an individual can self-reflect on their experiences and behavior at work [1]. Self-reflection can be defined as an individual’s attitude toward actively exploring and inspecting themselves internally, and this kind of attitude leads to a mature psychological mindset that can help them to find true wisdom in life. Self-reflective introspection influences not only problem-solving skills, individual attitude changes, and studying circumstances but also, afterward, informal learning within the workplace [2]. Several previous studies have shown high levels of stress, depression, and burnout among medical students [3, 4]. Self-reflection helps to improve their quality of life and ability to manage mental health [5–7]. Self-reflection is the main key to self-awareness and it may promote their self-healing psychologically [5, 6]. In relation to fostering self-reflection practice, it has been reported on the effectiveness of positive psychology and mindfulness-based intervention programs in medical schools [8–12].

Reflective writing can help learners become comfortable with a reflective process. Reflective writing is used for students in medical education to encourage them to self-reflect [13–15]. For at least the past decade, reflective writing has played a prominent role in medical education curricula [16–18]. Reflective journals offer a method of enabling students to self-reflect on their experiences. More specifically, in this approach, the learning method is expressed through writing, which students use to honestly engage in the reflective process. This approach promotes students’ critical thinking about the learning process while also enhancing their learning experience and management ability regarding that process [19]. Reflective writing represents a significant ’learning method’ through which to improve students’ reflective ability. Further, it offers both an important ’evaluation method’ through which to re-evaluate students’ learning process and an opportunity for students to re-evaluate themselves by reflecting on their learning experiences [20].

Reflective writing has always been required in various educational contexts in the field of medical education. It has been shown that reflective writing encourages students to learn self-directed learning methods and skills related to self-reflection [7]. Furthermore, skills such as video recording patient interviews and free, note-style self-reflection writing are carried out to this end. Self-reflection can occasionally be considered a troublesome task; on the other hand, it can also change individual awareness through several learning processes—practice, feedback, and self-reflection [21]. In addition, in PBL learning, reflective journals have a positive effect on problem solving, critical thinking, cooperative learning ability, and confidence [22]. Students are encouraged to apply their learning to real “problems” through the self-reflection process in PBL learning. This process enables students to gain a deep understanding of the reflected truth and an appreciation of its real value. Through self-reflection, they integrate new knowledge with existing knowledge, thus acquiring a more rounded, fully developed form of knowledge that they can use to assess both themselves and the problems they face [22].

This point was also observed in the reflective writing experience of reviewing online cases, and it was found that students learn about new topics and develop meta-cognition of their skills at the same time [23]. In another study, students experienced a sense of professional identity through reflective writing in a longitudinal integrated clerkship [24]. Students felt that the longitudinal narrative medicine-based portfolio curriculum, which involved a relationship with faculty mentors, was a safe space in terms of time and place; they experienced narratives and said that it was a moment of turning a mirror on oneself. They also said that they had an experience of moving through time [25]. This reflective writing experience was also found to be related to communication skills [26]. That is, through reflective writing, medical students can experience self-development including metacognition and communication skills.

As mentioned above, in the medical education field, future doctors must have reflective capacity as professionals, and approaches designed to apply reflective writing to medical education are being used in various educational contexts. However, in advance of its educational application, it is difficult to find studies concerning either the self-reflective writing levels of students or their opinions regarding writing reflective journals. Although the importance of self-reflection has been emphasized, studies have mostly focused on reflective activities concerning personal experiences in small group activities [18, 27, 28]. Fragmentary studies have also been performed, showing that reflective writing influences PBL, performance tests, and portfolios [16–18, 22]. Reflection is a valuable competency, but it is also perceived negatively by medical students. They may be to motivate poorly for self-reflection due to their excessive academic burden on the medical curriculum [29]. To overcome the students’ attitude about reflection and to provide them with educational intervention for self-reflection, it is necessary to first examine students’ level of reflective thinking and their perception and needs for reflection education. However, little has been reported about this in South Korea.

Therefore, the purpose of this study is to investigate how students’ understanding of reflection methods, their level of reflective thinking, and attitudes toward and perceptions of reflective essay writing change as their grades progress from pre-medical to the medical course. This study is a longitudinal observational study. In addition, we examined students’ need to be taught how to reflect. Through this study, it is possible to determine whether medical students continually develop their understanding and attitude toward reflection as they progress through grade levels. This will help to understand the necessity of reflection education as part of medical education.

The research questions are as follows:

1. How does students’ understanding of reflection change over time?
2. How does the reflective thinking level in writing change over time?
3. How do reflective writing attitudes and perception change over time?
4. How do students’ needs for reflective method education change over time?

## Materials and methods

### Sample

**Fig 1:**
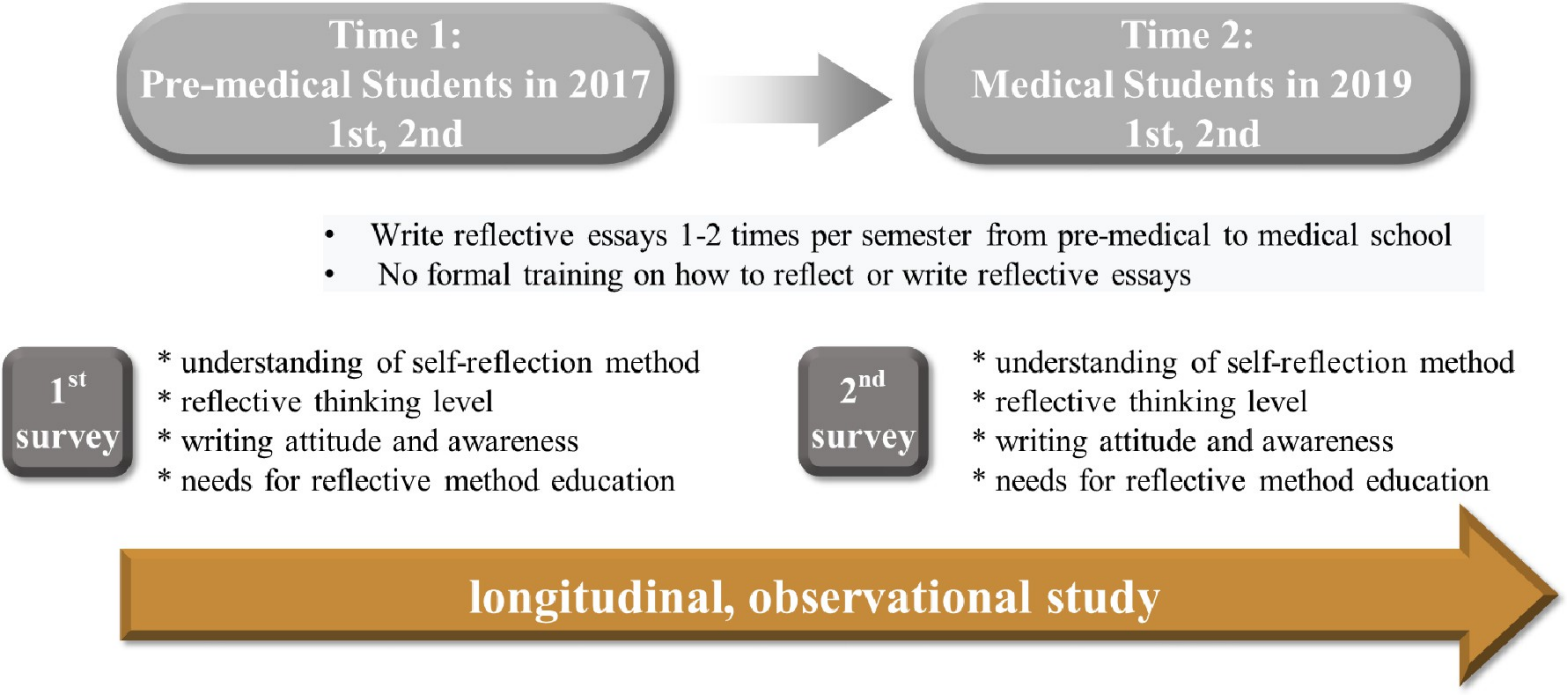
Research design.

This is a longitudinal and observational study (Fig 1). The participants in this study were 177 students at a medical university who were either first- or second-year pre-medical course students in 2017 and subsequently advanced to become either first- or second-year medical course students in 2019. The same test was performed with the same participants two years later. The number of students who responded in both instances was 137 (77.4% response rate). This cohort comprised 73 first-year students (53.3%) and 64 second-year students (46.7%). There were 87 male (63.5%) and 50 female (36.5%) students. These students had never received special education on how to write a reflective journal but had written one once or twice each semester. Results of the students’ responses were excluded from the analysis if they did not complete all the two surveys or did not respond seriously (for example, did not respond to the end).

### Instruments

To investigate students’ reflective thinking level in writing, questions on their understanding regarding self-reflection methods (1 question, “Do you know to some extent how to reflect and write?”) and writing attitude as well as on their knowledge of reflective journals (3 questions, e.g., what kind of attitude do you usually take when writing a journal with reflection?) and educational needs and methods regarding reflection (5 questions, e.g., Do you think that education on how to reflect and write a reflection journal for medical college students should be provided in the curriculum?) were included in the researchers’ self-developed questionnaire. The questionnaire developed by the researcher was used only after the content validity was verified by medical education experts. For students’ reflective thinking level in writing, the method developed by Galvez-Martin, Bowman, and Morrison [30] and subsequently adapted by Kwon [31] was used. Reflective thinking levels as reflected through writing were divided into seven phases: 0–2 low, 3–5 medium, and 6–7 high. Details of the seven phases are shown in Table 1 below.

**Table 1:** Reflective thinking level in writing.

Grade		Contents
Low	0	I described the contents in a short-answer format based only on my feelings.
	1	I simply described it as ’Learned about~’.
	2	I briefly summed up the contents and outlined my future learning plans.
Middle	3	I focused on my actions and feelings and described the future learning plan relatively, systematically, and logically.
	4	I approached it from an objective point of view, comparing it with others.
	5	I analyzed the contents of the class and found and described what could be applied.
High	6	I described it even considering other people’s perspectives
	7	I described knowledge, social perspective, and psycho-environmental factors in combination with my state and feelings and my reflected and suggested opinions.

### Data collection

The first survey in this study was concluded in 2017, and the second survey was conducted in 2019 with the same group. The researchers asked for consent and explained the specific purpose of the study before the survey, and if they agreed, written consent was signed and submitted. No compensation or payment was provided to them. The survey was conducted by grade group and without a time limit. It took approximately 15 minutes to complete. This study has been performed in accordance with the Declaration of Helsinki and has been approved by the Institutional Review Board of Pusan National University (IRB approval no. PNU IRB/2019_113_HR).

### Data analysis

The change in understanding of the self-reflection method and in reflective thinking level in writing according to students’ grade level was verified by χ2 test. The difference of change in reflective writing attitude and perception according to their grade level was verified by t-test. Frequency analysis was used to analyze their educational needs and methods regarding reflection.

## Results

### The change in understanding of the self-reflection method

To understand the change in the understanding of students’ self-reflection, the results of the surveys from both 2017 (first and second years of the pre-medical course) and 2019 (first and second years of the medical course) were examined closely. In terms of the change in understanding from the pre-medical course to the medical course, the number of students with a high level of understanding increased from 48.1% to 54.9%. The number of students with a medium level of understanding decreased slightly, from 29.3% to 27.1%, while the number of students with a low level of understanding decreased from 22.6% to 18.0%. This change did not show any meaningful difference (χ2 = 2.238, p = 0.692). Among high-understanding pre-medical students, there were 20 medium-understanding medical students (15%) and 9 low-understanding medical students (6.8%). Among the medium-understanding pre-medical students, there were 9 low-understanding medical students (6.8%). Consequently, the total number of students whose level of understanding decreased was 38. On the other hand, among medium-understanding pre-medical students, the number of medical students whose level of understanding increased was 22 (16.5%). Among low-understanding pre-medical students, there were 8 medium-understanding medical students (6%) and 16 high-understanding medical students (12.0%). Thus, the number of students whose level of understanding increased was 46, while the number of students who did not show any change was 49 (36.8%; Table 2).

**Table 2:** Changes in the understanding of self-reflection methods.

Level			Medical student (after 2 years; 2019)			Total
			High	Medium	Low	
Pre-medical Student (2017)	High	N (%)	35(26.3)	20(15.0)	9(6.8)	64(48.1)
	Medium	N (%)	22(16.5)	8(6.0)	9(6.8)	39(29.3)
	Low	N (%)	16(12.0)	8(6.0)	6(4.5)	30(22.6)
Total		N (%)	73(54.9)	36(27.1)	24(18.0)	133(100.0)

χ^2^ = 2.238, p = 0.692

High: I know how to reflect and write a reflection essay.

Medium: I know how to reflect but cannot write a reflection essay.

Low: I do not know how to reflect or write a reflection essay.

### The change in reflective thinking level

To analyze the change in students’ reflective thinking level, the results of the surveys from both 2017 (first and second years of the pre-medical course) and 2019 (first and second years of the medical course) were examined closely. In terms of the change in reflective thinking level from the pre-medical course to the medical course, the percentage of high-level students decreased from 10.3% to 5.9%, while that of medium-level students decreased from 22.1% to 16.9%. Meanwhile, that of low-level students increased from 67.6% to 77.2% (Table 3). However, this did not show any significant difference (χ^2^ = 8.003, p = 0.091).

**Table 3:** Changes in reflective thinking levels.

Level			Medical student (after 2 years; 2019)			Total
			High	Medium	Low	
Pre-medical Student (2017)	High	N (%)	3(37.5)	1(4.3)	10(9.5)	14(10.3)
	Medium	N (%)	2(25.0)	6(26.1)	22(21.0)	30(22.1)
	Low	N (%)	3(37.5)	16(69.6)	73(69.5)	92(67.6)
Total		N (%)	8(5.9)	23(16.9)	105(77.2)	136(100.)

χ^2^ = 8.003, p = 0.091

### The change in reflective writing attitude and perception

To analyze the change in students’ reflective writing attitude and perception, the results of the surveys from both 2017 (first and second years of the pre-medical course) and 2019 (first and second years of the medical course) were examined closely.

Students were asked to respond regarding their reflective writing attitude, ranging from “writing with sincerity with deep thought (5)” to “writing absentmindedly without deep thought (1)”in 5 Likert scale.

The average was significantly lower in the medical course (M±SD = 2.74±1.06) than in the pre-medical course (M±SD = 3.16±0.91) (t = 3.475, p = 0.001). The results show that students wrote more absentmindedly as they progressed further in their education. The perception that reflective writing was helpful for individual improvement decreased significantly during the medical course (M±SD = 2.62±1.07), in comparison to the pre-medical course (M = 3.20±0.77) (t = 4.931, p = 0.000). This shows that students’ belief that reflective writing was not helpful increased (Table 4).

**Table 4:** Changes in attitudes and perception regarding reflective writing.

Content	Time	N	M	SD	t	p
Active attitude in writing reflective essays	Pre-Med	136	3.16	0.913	3.475	0.001
	Med	136	2.74	1.061		
Degree of perception that writing a reflective essay is helpful for personal development	Pre-Med	137	3.20	0.768	4.931	0.000
	Med	137	2.62	1.072		

N: number, M: mean (5 Likert scale), SD: standard deviation, t: t test value, p: probability value

Medical students who answered that they wrote reflective essays absentmindedly were asked to provide a reason via an open question. In terms of the reason, they wrote the essays absentmindedly, 38 students (51,43%) answered that they put effort only into work submission, 19 students (27.14%) answered that it was meaningless, and 12 students (17.14%) answered that it was bothersome.

### Educational needs and methods regarding reflection

In terms of the necessity of reflective writing education, the average increased significantly during the medical course (M±SD = 3.42±1.07), compared to the pre-medical course (M±SD = 3.06±1.04) (t = -2.659, p<0.001). The need for education regarding reflective writing increased.

As a result of the needs assessment regarding reflection education, students responded that the appropriate period for this type of education was in the first year of both the pre-medical course (34.2%) and the medical course (56.2%), and the preferred length of education was no more than two hours (34.2%). Students’ preferred type of education was in the form of a lecture (34.2%), and their preferred content for this lecture regarded the importance of reflective essays and ways to write. In terms of writing style, freestyle writing was preferred (74.0%).

## Discussion

Medical students who learn how to reflect during university know how to develop clinical relationships with patients [32, 33]. Furthermore, self-reflection plays a successful role in integrating new knowledge with existing forms of knowledge. Self-reflection represents the essence of lifelong learning and helps solve complicated medical problems as a medical professional [34]. The best way to provide education regarding self-reflection is after understanding the reflection levels of students in medical courses. This study aimed to identify the types of changes that occur in students’ reflective thinking levels and writing attitudes. In addition, it investigated both the necessity of reflective education and different methods through which to provide such education. A discussion of the results is provided below.

As students’ progress through medical education, their level of understanding regarding reflection methods increases slightly. However, a statistically significant level of difference was not observed in this study. There was no improvement in students’ level of understanding regarding self-reflection methods level from the pre-medical course to the medical course. These results show that the method of self-reflection is not acquired and improved by students themselves. Self-reflection is closely related to feedback, and feedback is essential for an effective reflection [7]. Feedback leads to critical self-assessment to enhance performance and self-appraisal or self-mentoring techniques [35]. Therefore, this suggests that appropriate feedback from faculty or mentors is needed to improve students’ skills in self-reflection methods. Meanwhile, there was also no significant change in the level of reflective thinking in writing from the pre-medical to the medical course. The participants of this study had not received any prior education concerning how to write reflective journals. Considering this, these results may provide one suggestion in terms of teaching students how to write reflective journals in medical education. However, simply writing a reflective journal itself is insufficient in terms of teaching students how to self-reflect. Wald, Reis, and Borkan [36] state that reflective writing on its own does not improve an individual’s reflection quality or level. A previous study reported that the number of students writing critical reflection papers increased after a reflection workshop [37]. This suggests that education in reflective writing should be provided for students. As with the previous results, the role of the faculty is important, and an educational strategy is needed to improve the student’s reflection ability by providing feedback on the written reflection journal and inducing a deeper reflection.

Student’s attitude and perception of reflective writing were negatively changed. Students wrote reflective journals more absentmindedly during medical courses than in pre-medical courses. The reason for this was that they only placed meaning on their assessment submissions. One of the biggest challenges in teaching self-reflection in medical education is students’ low participation rate, which has previously been considered [38]. Because of this lack of motivation, medical students and even some professors are unaware of the importance of reflection in the learning process. It has been reported that in terms of reflection portfolio activity, students are pressed to complete the portfolio by focusing more on completion itself rather than on self-reflection. In addition, many students view reflection as an assessment they are obliged to complete [39] or as meaningless [40], and they do not want to dedicate time to self-reflection [41]. Despite the many advantages of self-reflection, students’ motivation to participate in this process is low. Medical students who are accustomed to summative evaluations often believe that self-reflection is overly time consuming [42]. Therefore, determining a way to facilitate students’ acceptance of reflection is extremely important. To ensure that the purpose of a reflective journal or portfolio extends beyond its use as an evaluation tool or imperative assessment, education designed to ensure that students perceive the meaning and necessity of reflection needs to be put in place beforehand. In addition, it should also be considered to provide students with a guide and checklist to understand their reflective level and competency.

In this study, the need for reflective journal writing education was shown to be higher in medical courses than in pre-medical courses. In terms of the period of education, the first year of the medical course is preferred, while for the type of education, a lecture of up to two hours is preferred. In the case of reflective journals, freestyle writing is preferred over a structured style. In advanced research, the preferred period and method are not mentioned, but the importance of self-reflection education is stressed as highly necessary in medical education curricula [42]. For instance, at the British Dundee Medical University, students were asked to write reflective journals and case reports as part of the curriculum after experiencing 100 clinical examples. As a strategy to encourage medical students to participate, professors should learn the advantages of reflection and how to evaluate it, while students should understand the advantages of reflection (personal professional improvement, story-telling skills, research techniques, academy writing, and communication skills development, etc.). Every medical university should develop a study module for reflection that is open to participation for every student.

Consequently, self-reflection will expand both in-depth learning and learning experiences, representing the only way to lead students from being novices to becoming experts. However, for various reasons, students do not participate in reflection in medical universities, and there is a lack of validity for evaluating reflection. Therefore, each medical college must develop an educational program and a feedback and evaluation system for student reflection that allows students to actively participate in experiences that can be reflected in the curriculum.

### Study limitations

We only studied a single sample of pre-clinical students from one medical school so its results and interpretations should be carefully applied to other students. By increasing more samples and accumulating further studies, the findings of the study should be generalized. Another limitation is the nature of self-reported questionnaire. So, we recommend that it is necessary to examine the change by comparing the reflective thinking level perceived by the students and the result by the faculty. Finally, during the increase of the grade, we examined the change in students’ level of reflective thinking in writing, without an educational intervention for reflective methods. In the future, it is necessary to examine the effect of the implementation of the educational intervention.

## Conclusions

There was no significant change in the level of understanding of self-reflection methods and the level of reflective thinking in writing from the pre-medical to the medical course. In the medical course, it was found that their attitude and perception of reflective writing were lower. Students are demanding the educational need for reflective writing education. As such, with the increase of grade level, self-reflective thinking does not naturally improve. Therefore, educational intervention is needed so that students can realize the importance of reflection and improve their reflective ability by learning how to reflect. In addition, it is necessary to provide students with specific guidance for self-reflection and to prepare an educational strategy for providing feedback by faculty.
